# Predicting Crash Injury Severity with Machine Learning Algorithm Synergized with Clustering Technique: A Promising Protocol

**DOI:** 10.3390/ijerph17155497

**Published:** 2020-07-30

**Authors:** Khaled Assi, Syed Masiur Rahman, Umer Mansoor, Nedal Ratrout

**Affiliations:** 1 Civil and Environmental Engineering Department, King Fahd University of Petroleum and Minerals, Dhahran 31261, Saudi Arabia; umerkhan190@gmail.com (U.M.); nratrout@kfupm.edu.sa (N.R.); 2 Center for Environment and Water, Research Institute, King Fahd University of Petroleum and Minerals, Dhahran 31261, Saudi Arabia; smrahman@kfupm.edu.sa

**Keywords:** crash injury severity, emergency management, feedforward neural networks (FNN), fuzzy c-means clustering (FCM), machine learning, support vector machines (SVM)

## Abstract

Predicting crash injury severity is a crucial constituent of reducing the consequences of traffic crashes. This study developed machine learning (ML) models to predict crash injury severity using 15 crash-related parameters. Separate ML models for each cluster were obtained using fuzzy c-means, which enhanced the predicting capability. Finally, four ML models were developed: feed-forward neural networks (FNN), support vector machine (SVM), fuzzy C-means clustering based feed-forward neural network (FNN-FCM), and fuzzy c-means based support vector machine (SVM-FCM). Features that were easily identified with little investigation on crash sites were used as an input so that the trauma center can predict the crash severity level based on the initial information provided from the crash site and prepare accordingly for the treatment of the victims. The input parameters mainly include vehicle attributes and road condition attributes. This study used the crash database of Great Britain for the years 2011–2016. A random sample of crashes representing each year was used considering the same share of severe and non-severe crashes. The models were compared based on injury severity prediction accuracy, sensitivity, precision, and harmonic mean of sensitivity and precision (i.e., F1 score). The SVM-FCM model outperformed the other developed models in terms of accuracy and F1 score in predicting the injury severity level of severe and non-severe crashes. This study concluded that the FCM clustering algorithm enhanced the prediction power of FNN and SVM models.

## 1. Introduction

### 1.1. Background

The number of traffic crashes and their victims has been a rising trend globally due to increases in population and motorization. Different factors involved in traffic crashes have a substantial effect on each other, thus making it difficult to individually consider any of the parameters when explaining the severity of traffic crashes. 

Traditionally, different statistical techniques have been employed to predict the severity of traffic crashes. Among those statistical models, ordered probit (OP) model [1,2,3,4,5], ordered logit (OL) model [6], multinomial logit (ML) model [7,8], and logistic regression (LR) model [9] are all widely used. Though these conventional techniques have been used for predicting crash severity, they suffer from a few inherent limitations. For example, the assumptions related to data distribution and a linear relationship between explanatory and dependent variables can be untrue and lead to inaccurate inferences [10,11]. To overcome these limitations, many ML techniques have been introduced to model crash severity [12]. These models include the Bayesian network (BN) model [13,14], regression tree cart model [15,16], and artificial neural networks (ANN) [10,17]. 

### 1.2. Application of Statistical Models in Crash Severity Prediction

The approach for modeling crash severity involves statistical modeling considering severity as a dependent variable while driver, vehicle, roadway, and environmental factors as independent variables. Regression techniques like logit and probit have been used to model traffic crash severity [18,19]. Some studies used binary probit and logit models [1,20,21] for modeling two levels of crash severity, while others used multinomial logit and probit for modeling multiple severity levels [22,23]. Several advanced models have been developed to account for the ordinal nature heterogeneity and correlation among variables in traffic crash data. These models include Bayesian hierarchical [24], ordered probit and logit models [2,25,26,27], nested logit models [23,28,29], and their combined versions [30,31].

An ordered probit model was developed to predict the severity of road crashes [32]. Many contributing factors related to human, vehicle, and roadway were examined to see their effects on crash severity. The study concluded that crash severity was affected significantly by area type (urban or rural) and gender. Kockelman and Kweon [2] developed probit models for all crash types of single vehicles and two vehicles. Sports cars and pickups were involved in serious crashes compared to passenger cars, while young male drivers driving at lower speeds in newer cars were involved in less severe crashes. Chen et al. [33] conducted a study to identify significant factors affecting the severity of truck-involved crashes using probit models. It was found that gender, age, time of day, weather conditions, and many other factors significantly affect crash severity. Hu et al. [34] developed a logit model to investigate the main factors influencing crash severity at railroad junctions. It was found that the number of daily trains, number of daily trucks, obstacle detection device, and approaching crossing marking significantly affect crash severity. A comparison study was conducted between the Bayesian network and regression modeling for crash severity prediction in china [35], where the Bayesian network outperformed regression modeling in crash severity prediction. 

Al-Ghamdi [9] used logistic regression to identify variables contributing to crash severity in Riyadh, Saudi Arabia. The most significant variables affecting the crash severity were the crash cause and crash location. Multiple logistic regression (MLP) model forecasted the crash severity on expressways in Thailand and concluded that traffic speed is the most significant variable affecting the severity level [36]. A logistic regression model was developed to study and predict roadway and environmental effects on traffic crash severity in the USA [37]. Road alignment, light condition, speed limit, class of road, location, and pavement condition contribute significantly to the crash severity. 

### 1.3. Application of Machine Learning Models in Crash Severity Prediction

Without the assumptions of statistical models, several ML models have been employed for traffic crash severity prediction. ML algorithms can model the non-linear relationship crash severity and related factors. Sameen and Pradhan [38] predicted the injury severity of traffic crashes using recurrent neural networks (RNN) in Malaysia. It was found that RNN is superior, with an accuracy of 71.77% followed by multilayer perceptron neural network (MLP) and Bayesian logistic regression (BLS) with an accuracy of 65.48% and 58.30%, respectively. Similarly, the performance of the ANN and OP model were compared [39]. Fuzzy adaptive resonance theory (ART) and MLP were used to examine crash severity. The study revealed that MLP performed better, with an accuracy of 73.6%, whereas ART showed a classification accuracy of 70.6%. The results of the OP model were the least accurate, having an accuracy of 61.7%. ANN was used for crash severity prediction using 6 years of crash data for Abu Dhabi. The results of ANN were compared to the OP model and the ANN outperformed the OP model in terms of accuracy [40]. Similarly, ANN was used to predict the crash severity at a signalized intersection in Central Florida, USA. MLP and ART neural networks were compared based on accuracy and MLP performed better than ART [17].

Along with neural networks, many other ML models have been used for crash severity prediction. A study adopted ANN, SVM, decision tree (DT), and LR models to predict severity [41]. SVM showed the most accurate prediction followed by DT, ANN, and LR, respectively. A multi-objective genetic algorithm was compared with ANN, SVM, and DT for predicting crash severity in the capital of Iran. The technique was more accurate compared to ANN, SVM, and DT [42]. Zhang et al. [43] compared ML and statistical techniques for crash severity prediction. The study concluded that the ML techniques, although suffering from over-fitting issues, outperformed the classical statistical techniques in terms of prediction accuracy.

Similarly, a study was conducted to predict the traffic crash severity using ANN, genetic algorithm (GA), combined genetic algorithm (CGA), and pattern search (PS) methods, and their performance was compared [44]. The study revealed that ANN outperformed the other three methods having an R-value of 0.87, while an R-value of 0.79 was calculated for GA and PS methods. Li et al. [45] used a combination of GA and ANN to examine the factors affecting the crash severity in Washington, USA. The authors combined the GA with ANN architecture to enhance the efficiency of searching for the significant variables. It was found that driver conduct, vehicle action, roadway surface condition, driver restraint, and age significantly affect the crash severity. A deep learning-based convolutional neural network (CNN) was employed for predicting traffic crash severity. CNN was compared with several statistical techniques like LR and ML techniques like SVM and ANN. The study revealed that the CNN model performed better than all of the other techniques [46].

In the preceding sections (Section 1.2 and Section 1.3), we presented the studies related to traffic crash severity prediction. Many statistical techniques have been implemented by researchers followed by modern ML techniques like ANN, SVM, DT, and GA. As a general conclusion, we noticed that ML techniques outperformed conventional crash severity prediction techniques, which was also evident in most studies. 

### 1.4. Artificial Neural Networks

First introduced in the 1960s, the ANN can solve many complex analytical problems. It is a biologically-motivated machine learning tool that captures and represents extremely complex non-linear relationships existing in real work data sets. It works by mimicking the neurological functions of the human brain, just as neurons stimulate and react to a situation in the human brain. It can predict the outcome of an observation based on the pattern caught from historical data after carrying put a training procedure [41]. An artificial neuron is the basic unit of a neural network. Input nodes transfer the knowledge to a neuron, which is processed internally to produce a response. ANN is processed in two steps: the first step is the linear combination of input values and then the obtained results are used as an argument for non-linear activation function. Each connection has a weight assigned to it and the activation function is differentiable [47].

**Figure 1:**
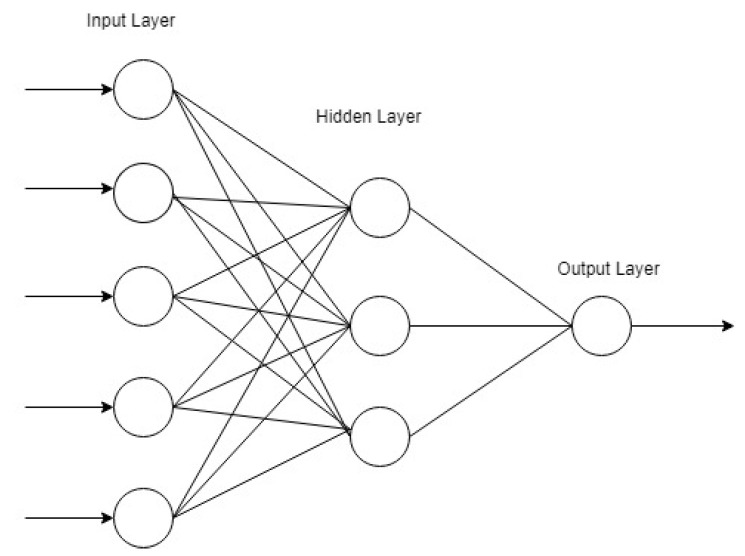
Typical feed-forward neural network.

The network architecture is defined by neurons organization. An example of a neural network is presented in Figure 1. The architecture consists of an input layer, a hidden layer, and an output layer. The input, hidden, and output layers consist of five, three, and one neuron, respectively. The output of one layer is utilized as input to the next layer. The activation function in the neuron combines the inputs by multiplying with the corresponding weights. There is also a bias component in each neuron. Optimization methods are used to estimate the weights of the input (“called the training of network”) by minimizing the loss function. Several training algorithms are available; one of them is backpropagation, which is based on a gradient descent technique for parameter estimation [47].

### 1.5. Support Vector Machine

SVM is an extensively used ML technique. SVM works under the principle of supervised learning that uses labeled training data to deliver input and output functions, just like neural networks. Input and output functions are related to each other either by classification or regression type functions. Just like neural networks, both classification and regression prediction models can be handled by SVM [41,48]. 

SVM is a supervised ML technique that does not require any assumption about the data distribution. Labeled data sets are presented in the original formulation and the main aim of the SVM training algorithm is to look for a hyperplane, thus separating data set into discrete sets consistent with the training examples. The decision boundary is often referred by the term optimal separation hyperplane, which minimizes the misclassifications found in the training stage. Classifier with optimal decision boundary is found by the iterative process of learning, which separates the training pattern and the simulation data under the same configuration. The simplest form of SVM is a linear binary classifier in which a class from one of the two labels is assigned to a given test sample [49].

**Figure 2:**
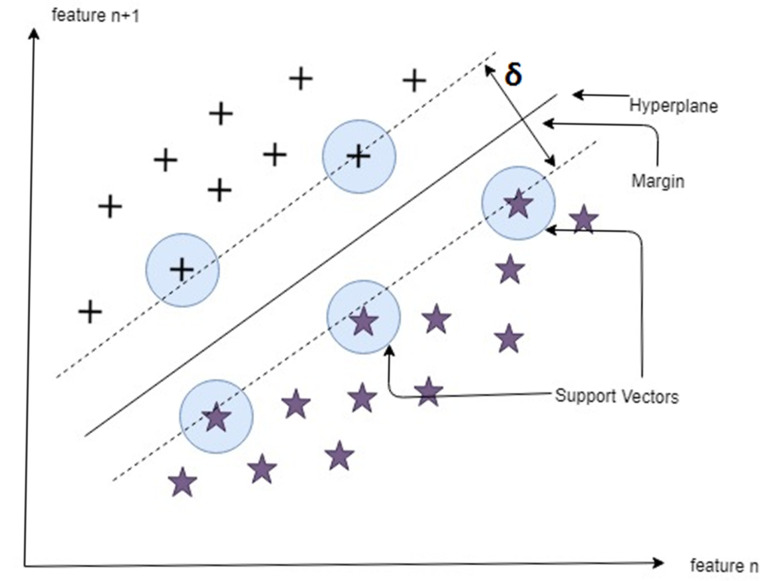
Maximum separation hyperplane.

SVM is a comparatively strong machine learning technique in many fields due to its theoretical framework, which can perform more efficiently for noise mixed data compared to the conventional chaotic local models. This technique is also valid for small data sets along with global optimization and better generalization performance. Minimizing general error and maximizing the margin is the primary intent of SVM by separating hyperplane between two classes [50]. Figure 2 explains the general idea of a support vector machine. It has two classes of support vectors separated by a hyperplane. The points which are closest to the hyperplane are called support vectors. Support vectors are critical points that, if eliminated, would alter the position of the hyperplane. The hyperplane is a simple line classifying the data. Higher the margin between support vectors, more confident we are about the correct classification of data by the hyperplane.

### 1.6. Fuzzy C-Means Clustering

Fuzzy c-means clustering algorithm (FCM) was introduced by Dunn in 1973 [51] and enhanced by Bezdeq in 1981 [52], and it partitions a dataset into two or more clusters. A process of subdividing data set into subsets (clusters) is known as cluster analysis. Clusters are pairwise disjointed and reproduce the dataset upon merging. The FCM cluster technique is the extension of the K-means method. It repeatedly searches for a set of fuzzy clusters and associated clusters centers that provide the best representation of data set structure. The algorithm is user-dependent as the user has to state the number of clusters for cluster analysis. It divides the data set into clusters by reducing the sum of squared error within each group [53]. 

In this clustering algorithm, each cluster can be considered as a fuzzy set, and each training vector assigns membership grade to a cluster, which is measured by a membership function [54]. In FCM clustering, unlike hard C-means, each training vector belongs to multiple clusters [55]. In the FCM algorithm, the partitioning assigns each training vector to a cluster with the degree of belongingness varying from 0 to 1. It minimizes an objective function while partitioning the training vectors. The membership matrix U lies within 0 and 1, and the total membership grades of a training vector to all clusters is 1. The relevant expressions follow.

Summation of membership grades,

(1) $$\overset{c}{\underset{i=1}{\sum }}u_{ij}=1$$

Objective function,

(2) $$\;J_{m}=\overset{c}{\underset{i=1}{\sum }}\overset{N}{\underset{j=1}{\sum }}u_{ij}^{m}\|x_{j}-c_{i}\|{}^{2}$$

Calculated cluster center,

(3) $$c_{i}=\frac{\sum_{j=1}^{N}u_{ij}^{m}X_{j}}{\sum_{j=1}^{N}u_{ij}^{m}}$$

and calculated membership grade,

(4) $$u_{ij}=\frac{1}{\sum_{k=1}^{c}{\left(\frac{\left\|x_{j}-c_{i}\right\|}{\left\|x_{j}-c_{k}\right\|}\right)}^{\frac{2}{\left(m-1\right)}}}$$

where *j* is 1, …, *N; c* is the number of clusters; *m* is any real number greater than 1 known as fuzzy exponent; *u_ij_* is the membership value of *x_j_* for the cluster I; *x_j_* is the *j^th^* of d-dimensional measured data; *c_i_* is the d-dimension center of the cluster; and ||*|| is the Euclidean distance between any training vector and the center.

### 1.7. Study Objectives

The objective of this paper was to predict crash severity using information that could be easily identified with a little investigation on crash sites. Such knowledge enables trauma centers to predict injury severity accordingly, dispatch the properly equipped emergency vehicle, and then guide it to the nearest hospital. Empowering trauma centers with such ability can improve traffic safety especially in remote areas where emergency services are scarce. The parameters used in this paper mainly include vehicle attributes and road condition attributes.

The main goal of traffic crash injury severity modeling is to comprehend the relationship between crash injury severity and many contributing factors like human characteristics, vehicle characteristics, roadway characteristics, and environmental characteristics. The aim of this study is to investigate the role of clustering algorithms in the enhancement of the performance of machine learning models, such as ANN and SVM. It will create a new paradigm in developing machine learning models for crash severity prediction.

### 1.8. Outline

The rest of the paper is organized as follows: Section 2 explains the dataset used in model development. The methodology followed in models’ development and validation is described in Section 3. Section 4 presents results and discussion. Section 5 outlines the main conclusions and explains the limitations and recommendations for future study.

## 2. Data Set Description

**Figure 3:**
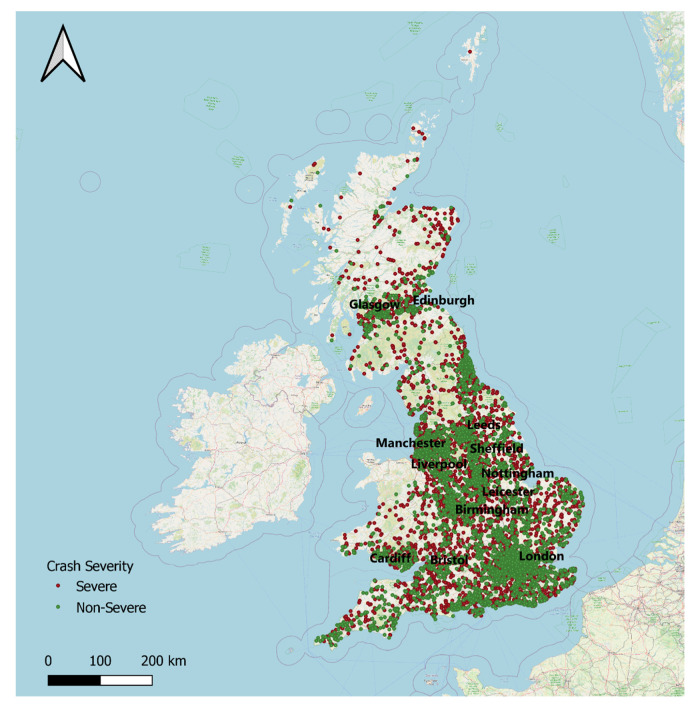
Spatial distribution of crashes in Great Britain (GB).

The crash dataset in Great Britain, UK, between 2011–2016 was used in this study. A random sample of crashes representing each year was used for analysis. A randomized class balancing procedure [56] was followed in selecting the sample. In this procedure, a sample was randomly selected in such a way that each class had an equal number of events in the dataset to make sure that the sample was balanced without any bias towards a specific severity level. The spatial distribution of crashes for the sample is illustrated in Figure 3, representing both severe and non-severe crashes in the Great Britain (GB). From this figure, it is evident that crashes were highly crowded in London. The selected input attributes that belonged to vehicle, road, and crash are shown in Table 1. Features that could be easily identified with a little investigation on crash sites were used as an input so that the trauma center could predict the injury severity level based on the initial information provided and prepare accordingly for the treatment of the victims. The output variable was the crash severity (i.e., severe or non-severe). A crash with at least one fatality or serious injury was considered severe. The severity was reported in three levels in the original data set: fatal, serious, and slight, with a proportion of 1.5%, 15.5%, and 83%, respectively. In this research, the fatal and serious crashes were merged under one severity class, i.e., severe crash. In contrast, slight crashes were considered a non-severe crash. It should be mentioned that the following types of injuries were considered serious injuries:

- The injury that causes a person to be detained in hospital as an in-patient for an extended period and which may have required surgery.
- An injury that will have lasting or even permanent implications for the injured person and that will have an impact upon their ability to work or which involve a change to their level of independence.
- An injury that causes death 30 or more days after the accident.

**Figure 4:**
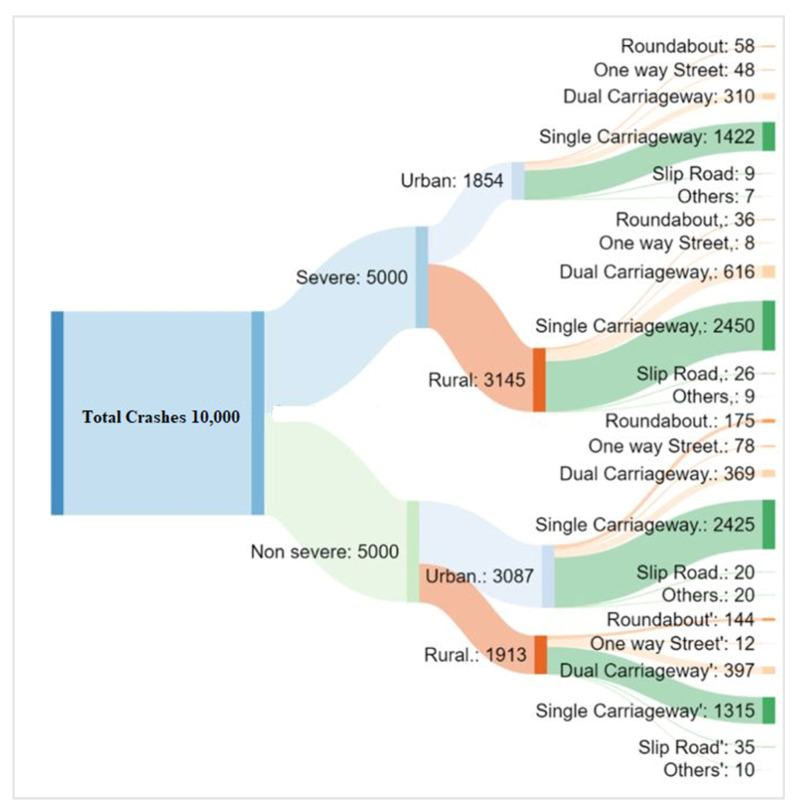
Distribution of crashes for area type and road type.

Before developing the ML models, the dataset was preprocessed to make sure that the crash dataset was clean and valid. The crash distribution with respect to area type and road type was illustrated in Figure 4. Figure 4 explains that a higher number of crashes in rural areas was severe, while urban areas mostly encountered non-severe crashes. This trend was obvious due to higher speeds in rural areas leading to severe crashes, while the speeds in urban areas are comparatively slower, minimizing the possibility of severe crashes. Most crashes occurred on a single carriageway since two-way traffic is not separated by a median, thus leading to higher chances of crashes.

## 3. Model Development

In this study, the MATLAB Environment (MathWorks, Natick, MA, USA) was used throughout the analysis to develop and validate all models using the variables mentioned in Table 1 as inputs. Four ML models were considered in the study: feed-forward neural networks (FNN), support vector machine (SVM), fuzzy c-means clustering based feed-forward neural network (FNN-FCM), and fuzzy c-means based support vector machine (SVM-FCM). The models were trained and tested based on the same dataset for unbiased comparison. Description of the developed models and the resulted accuracies are provided in the following sections. For training purposes for all developed models, 70% of the dataset (7000 crashes) was used while the remaining 30% were used for testing (3000 crashes).

### 3.1. Feedforward Neural Networks

The neural network model was developed in a systematic procedure for predicting crash severity. A systematic trial and error approach followed, starting with a small number of hidden layers and then built larger until an acceptable accuracy was achieved without causing overfitting. Different training algorithms were tested in building the FNN model such as Levenberg–Marquardt (LM), resilient backpropagation (RB), scaled conjugate gradient (SCG), BFGS quasi-Newton (BFG), Bayesian regularization backpropagation (BR), and variable learning rate backpropagation (GDX). 

LM learning algorithm resulted in the highest classification accuracy compared to the other training algorithms. The optimum topology for the FNN model with the highest classification accuracy on the testing set was found to be two hidden layers with 32 and 2 neurons in the first and second layers respectively using hyperbolic tangent sigmoid and softmax activation functions for the first layer and second layer, respectively. The learning rate, goal, and number of epochs of the network were 0.00002, 0.000001, and 100, respectively. The loss function was the mean squared error. 

### 3.2. Support Vector Machine

A SVM model was developed to predict crash severity using driver, roadway, and crash characteristics as input parameters. In developing any SVM model, the values of the penalty parameter C and insensitivity zone ε should be determined. The trade-off between training error and model complexity was controlled by parameter C. Insensitivity zone ε affected the complexity and generalization capability of the SVM model as it controlled the smoothness of SVM response and the number of support vectors. 

The optimization routine of the classifier was based on the iterative single data algorithm proposed by Kecman et al. [57]. A systematic trial and error procedure was followed to determine the values of C and ε. After many trial and error experiments, the best values of C and ε were found to be 15 and 150, respectively. One of the important parameters that can affect the classification accuracy of the SVM model is the kernel function. Many kernel functions were tried in developing the SVM model, such as polynomial kernel, Gaussian kernel, Gaussian radial basis function, Laplace RBF kernel, hyperbolic tangent kernel, sigmoid kernel, and ANOVA radial basis kernel. In this study, the Gaussian radial basis function was adopted as it resulted in the highest classification accuracy compared to the other functions. The kernel scale parameter was 15. The classifier divided all elements of the predictor matrix by kernel scale value. A box constraint controlled the maximum penalty enacted on margin-violating observations, which prevented overfitting. The classifier generated fewer support vectors for a high value of box constraint. The value of the box constraint was 200 in this modeling exercise. The resulted accuracy of the developed SVM model found to be 73%.

### 3.3. FCM-Based FNN and SVM

The traffic crash data were divided into different clusters using the FCM clustering algorithm to investigate the effect of clustering on the classification accuracies of FNN models. The exponent, number of iterations, and desired improvement in the objective function for the FCM were 2, 100, and 0.0001, respectively. The learning algorithm of the FNN was LM. In all the cases, hyperbolic tangent sigmoid activation functions were used for both layers. The number of neurons, learning rate, goal, and number of epochs of the FNN models varied depending on clusters. The loss function was the mean squared error for all models. 

The FCM clustering algorithm was also adopted to investigate the effect of clustering on the classification accuracies of SVM models. The exponent, number of iterations, and the desired improvement in the objective function for the FCM were 3, 100, and 0.0001, respectively. The optimization routine of the classifier was based on the iterative single data algorithm. All of the predictor variables were normalized using. The kernel scale parameter varied between 5 and 7 depending on clusters. The value of box constraint was 150 for all classifiers.

A systematic approach was followed to determine the optimal number of clusters based on prediction accuracy, as shown in Table 2.

It can be observed from Table 2 that the optimal number of clusters of FNN-FCM and SVM-FCM were 2 and 4 clusters, respectively. For the FNN-FCM model, one ANN model was developed for each cluster with a total of 2 FNN models. The optimum topology for each model was found following the same procedure explained in Section 3.1. After training the models, all crashes in the testing dataset were distributed between the two clusters and the corresponding FNN model was executed to predict the severity of each crash. The overall testing accuracy for FNN combined with FCM found to be 71.8%, which represents a slight improvement compared to testing accuracy of FNN without clustering which was found to be 70.1%. 

For the SVM-FCM model, one SVM model was developed for each cluster with a total of four SVM models and the same testing procedure was repeated. The overall testing accuracy for SVM combined with FCM found to be 74.2%, which indicates that combining FCM with SVM had higher accuracy when compared with SVM. 

## 4. Results and Discussion

**Figure 5:**
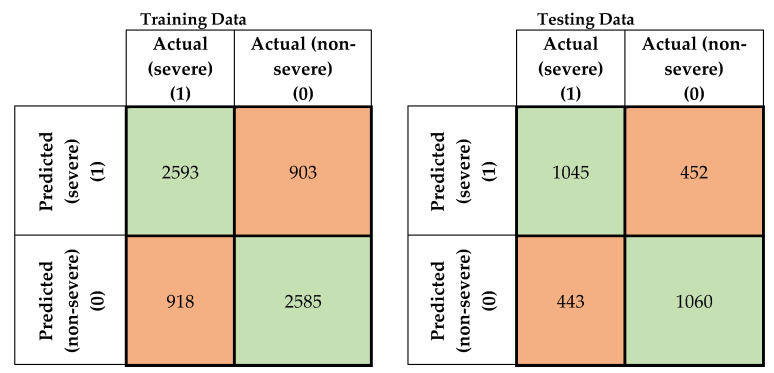
Confusion matrices for feed-forward neural network (FNN) model (training and testing data).

**Figure 6:**
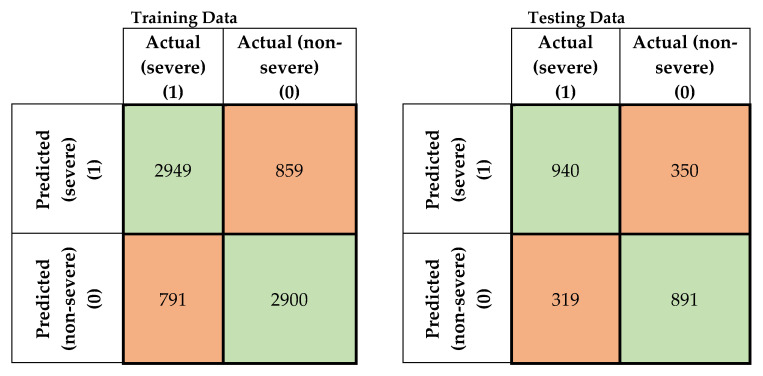
Confusion matrix for the support vector machine (SVM) model (training and testing data).

**Figure 7:**
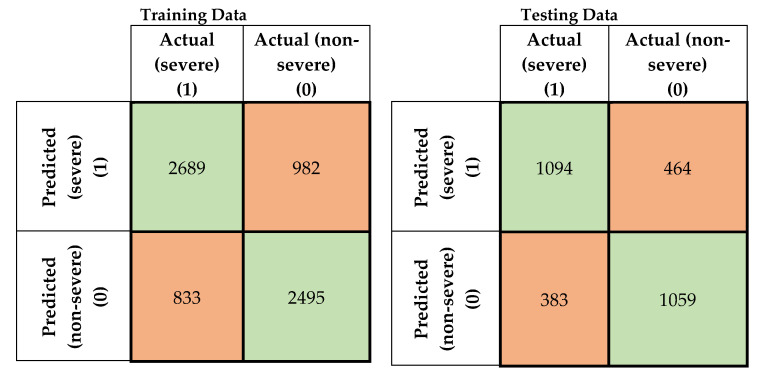
Confusion matrices for FNN combined with fuzzy c-means (FCM) clustering (training and testing data).

**Figure 8:**
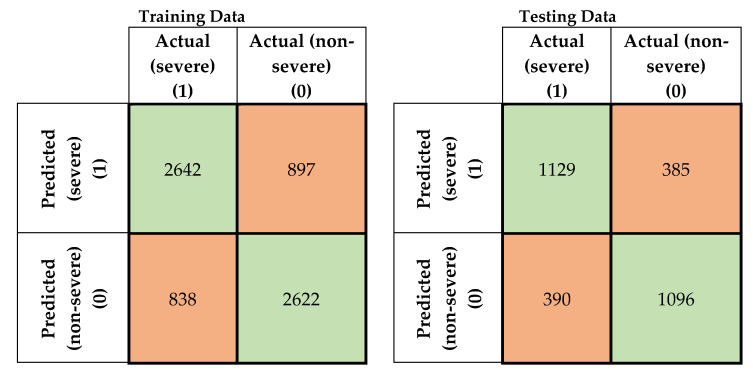
Confusion matrices for SVM combined with FCM (training and testing data).

In this study, four machine-learning models were developed to predict the severity of traffic crashes. Two severity levels were considered in this study (severe and non-severe). To evaluate the developed models, confusion matrices for the training and testing data for the developed models were prepared, as shown in Figure 5, Figure 6, Figure 7 and Figure 8. To evaluate the developed models, confusion matrices for the training, and testing data for the developed models were prepared (Figure 5, Figure 6, Figure 7 and Figure 8). A confusion matrix is a simple table that is used usually to describe the performance of classification models on test data for which the true values are known. The diagonals in Figure 5, Figure 6, Figure 7 and Figure 8 running from NW to SE represent true predictions. On the contrary, diagonals running NE to SW depict false predictions. Several statistics are calculated from the confusion matrices, namely classification accuracy, sensitivity, precision, and F1 score, which are explained in Equations (5)–(10).

The classification accuracy is defined as the number of correct predictions divided by the total number of observations and can be explained using Equation (5).

(5) $$\begin{array}{c} \mathrm{Classification}\;\mathrm{Accuracy}=\frac{\mathrm{Number}\;\mathrm{of}\;\mathrm{crashes}\;\mathrm{correctly}\;\mathrm{predicted}\;\mathrm{as}\;\mathrm{severe}/\mathrm{non}-\mathrm{sever}}{\mathrm{Total}\;\mathrm{number}\;\mathrm{of}\;\mathrm{crashes}} \end{array}$$

The classification accuracy results revealed that combining the clustering technique with FNN could enhance the prediction accuracy of FNN slightly. Combining FCM with SVM had higher accuracy when compared with SVM only. Moreover, based on the classification accuracy results, SVM-FCM models outperformed the other developed models. Nevertheless, using classification accuracy as the only metric to compare between the crash severity prediction models is misleading and not enough, as sometimes the developed model is biased towards a specific severity level.

In crash severity prediction models, prediction of crash severity is correctly critical and important as the actions required for sever crashes are different when compared with those of non-sever crashes. Hence, more metrics such as sensitivity and precision were considered in this study to investigate the capability of the developed models in predicting the crash severity. Sensitivity can be defined as the ratio of the crashes correctly predicted as sever/non-sever crashes to the total number of actual sever/non-sever crashes and can be expressed using Equations (6) and (7).

(6) $$\mathrm{Sensitivity}\;\left(\mathrm{severe}\;\mathrm{crashes}\right)=\frac{\mathrm{Number}\;\mathrm{of}\;\mathrm{crashes}\;\mathrm{correctly}\;\mathrm{predicted}\;\mathrm{as}\;\mathrm{severe}}{\mathrm{Total}\;\mathrm{actual}\;\mathrm{severe}\;\mathrm{crashes}}$$

(7) $$\mathrm{Sensitivity}\;\left(\mathrm{non}-\mathrm{severe}\;\mathrm{crashes}\right)=\frac{\mathrm{Number}\;\mathrm{of}\;\mathrm{crashes}\;\mathrm{correctly}\;\mathrm{predicted}\;\mathrm{as}\;\mathrm{non}-\mathrm{severe}}{\mathrm{Total}\;\mathrm{actual}\;\mathrm{non}-\mathrm{severe}\;\mathrm{crashes}}$$

Precision is the ratio of the crashes correctly predicted as sever/non-sever crashes to the total number of predicted sever/non-sever crashes and can be expressed using Equations (8) and (9).

(8) $$\mathrm{Precision}\;\left(\mathrm{severe}\right)=\frac{\mathrm{Number}\;\mathrm{of}\;\mathrm{crashes}\;\mathrm{correctly}\;\mathrm{predicted}\;\mathrm{as}\;\mathrm{severe}}{\mathrm{Total}\;\mathrm{predicted}\;\mathrm{severe}\;\mathrm{crashes}}$$

(9) $$\mathrm{Precision}\;\left(\mathrm{non}-\mathrm{severe}\right)=\frac{\mathrm{Number}\;\mathrm{of}\;\mathrm{crashes}\;\mathrm{correctly}\;\mathrm{predicted}\;\mathrm{as}\;\mathrm{non}-\mathrm{severe}}{\;\mathrm{Total}\;\mathrm{predicted}\;\mathrm{non}-\mathrm{severe}\;\mathrm{crashes}}$$

The best model has the maximum values of both sensitivity and precision. Sometimes, choosing the best classifier based on maximizing two parameters is confusing and not an easy task. Hence, for convenience, the harmonic mean of sensitivity and precision (F1 score) was introduced, which is expressed in Equation (10) [58].

(10) $$F1\;\mathrm{score}=\frac{2\times \mathrm{Precision}\times \mathrm{Sensitivity}}{\left(\mathrm{Precision}+\mathrm{Sensitivity}\right)}$$

**Figure 9:**
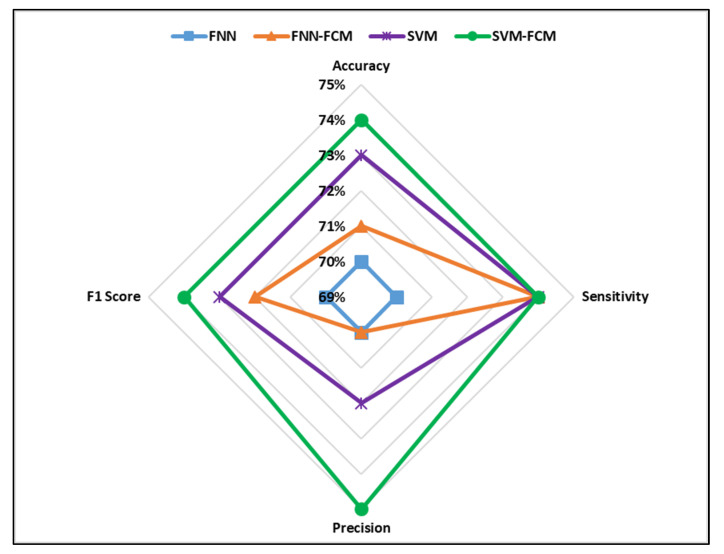
Performance measures of the developed models (severe crashes).

**Figure 10:**
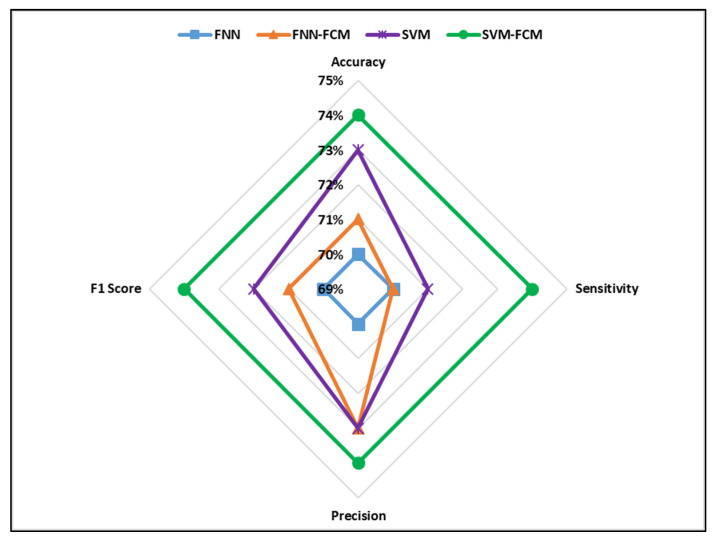
Performance measures of the developed models (non-severe crashes).

The values of accuracy, sensitivity, precision, and F1 score for all developed classifiers are shown in Figure 9 and Figure 10. 

The comparative analysis showed that the SVM-FCM model performed better than the other developed models based on accuracy and F1 score in predicting severe and non-severe accidents. Moreover, it can be observed that FNN had the least accuracy and F1 score values. Furthermore, the results revealed that introducing the FCM clustering algorithm enhanced the prediction power of FNN and SVM models. The prediction accuracies of clustering based models developed in this study were higher than ML models developed in previous studies for traffic crash severity prediction [38,39,43].

## 5. Conclusions

This study focused on predicting traffic crash severity by employing 15 crash-related parameters in four machine learning models: FNN, SVM, FCM clustering-based FNN, and FCM-based SVM. The models were developed based on the GB crash database data from 2011–2016. A random sample of crashes representing each year was used for analysis. Driver attributes, vehicle attributes, and road condition attributes were used as inputs when developing the models. Two severity levels were considered in this study: severe crashes and non-severe crashes. The severe crash was defined as the crash with at least one serious injury or a fatality. The severity prediction power of the developed models was evaluated using four measures of effectiveness: accuracy, sensitivity, precision, and the harmonic mean of sensitivity and precision (F1 score). The SVM-FCM model outperformed the other developed models in terms of accuracy and F1 score in predicting the severity level of severe and non-severe crashes. The FNN had the least accuracy and F1 score values. This study concluded that the FCM clustering algorithm enhanced the prediction power of FNN and SVM models. 

### Limitations and Future Study

The randomized class balancing procedure was used in this study to solve the problem of traffic crash imbalanced dataset. The authors could have used other advanced approaches to handle imbalance dataset issue. Moreover, the application of the developed models in developing countries might face challenges due to scarcity of the data. 

This work inspired the authors to think of simplifying the developed model in the future by eliminating some of the predicting variables. Although this is expected to reduce the model accuracy, it might make it agile enough to be utilized in other countries especially developing ones where traffic crash data is usually scarce.

## Figures and Tables

**Table 1:** Crash related factors.

Input Variables	Data Type	No. of Categories
Vehicle attributes		
Number of vehicles involved	Numeric	-
Vehicle type	Nominal	12
Road condition attributes		
Road type	Nominal	5
Junction type	Nominal	9
Junction control	Nominal	5
Light	Nominal	5
Weather	Nominal	9
Road surface condition	Nominal	7
Area type	Nominal	2
Speed limit	Numeric	-
Road class	Nominal	6
Crash attributes		
Number of causalities	Numeric	-
Day of the week	Numeric	7

**Table 2:** Accuracy results using different number of clusters.

No. of Clusters	FNN-FCM^1^ Testing Accuracy (%)	SVM-FCM^2^ Testing Accuracy (%)
1	70.0	73.0
2	71.8	72.2
3	71.0	73.0
4	70.2	74.2
5	67.9	72.1

^1^FNN-FCM: fuzzy c-means clustering based feed-forward neural network. ^2^SVM-FCM: fuzzy c-means clustering based support vector machine.
